# Cutaneous melanoma in older patients

**DOI:** 10.1186/s12877-024-04806-8

**Published:** 2024-03-06

**Authors:** Alessandra Buja, Massimo Rugge, Chiara Trevisiol, Anna Zanovello, Alessandra Rosalba Brazzale, Manuel Zorzi, Antonella Vecchiato, Paolo Del Fiore, Saveria Tropea, Marco Rastrelli, Carlo Riccardo Rossi, Simone Mocellin

**Affiliations:** 1 Hygiene and Public Health Unit, Laboratory of Health Care Services and Health Promotion Evaluation, Department of Cardiologic, Vascular and Thoracic Sciences, and Public Health, University of Padua, Via Loredan, 18, 35131 Padua, Italy; 2 Pathology and Cytopathology Unit, Department of Medicine - DIMED, University of Padua, Padua, Italy; 3 Veneto Tumour Registry (RTV), Azienda Zero, Padua, Italy; 4 Soft-Tissue, Peritoneum and Melanoma Surgical Oncology Unit, Veneto Institute of Oncology IOV-IRCCS, Padua, Italy; 5 Department of Statistical Sciences, University of Padua, Padua, Italy; 6 Department of Surgery, Oncology and Gastroenterology - DISCOG, University of Padua, Padua, Italy

**Keywords:** Melanoma, Age, Old, Histopathological characteristics, Clinical indicators, Cohort study

## Abstract

**Background:**

In industrialized countries, the aging population is steadily rising. The incidence of cutaneous malignant melanoma (CMM) is highest in old people. This study focuses on the clinicopathological profile of CMM and indicators of diagnostic-therapeutic performance in older patients.

**Methods:**

This retrospective population-based cohort study included 1,368 incident CMM, as recorded in 2017 by the Regional Veneto Cancer Registry (Northeast Italy). Older subjects were defined as ≥ 80, old as 65–79, and adults as < 65 years of age. The strength of association between pairs of variables was tested by Cramer’s-V. Using age groups as the dependent variable, ordered logistic regression was fitted using the clinicopathological CMM profiles as covariates. In each of the three age-groups, the indicators of clinical performance were computed using the Clopper-Pearson exact method.

**Results:**

Compared to patients aged younger than 80 years (1,187), CMM in older patients (181; 13.2%) featured different CMM topography, a higher prevalence of ulcers (43.3% *versus* 12.7%; *p* < 0.001), a higher Breslow index (*p* < 0.001), a lower prevalence of tumor-infiltrating lymphocytes (64.4% *versus* 76.5%, *p* < 0.01), and a more advanced pTNM stage at clinical presentation (*p* < 0.001). Elderly patients with a positive sentinel-lymph node less frequently underwent sentinel- lymph node biopsy and lymphadenectomy (60.0% *versus* 94.2%, and 44.4% *versus* 85.5%, respectively; *p* < 0.001).

**Conclusions:**

In older CMM patients, the clinicopathological presentation of CMM shows a distinctive profile. The present results provide critical information to optimize secondary prevention strategies and refine diagnostic-therapeutic procedures tailored to older patients.

## Introduction

Cutaneous malignant melanoma (CMM) accounts for less than 10% of skin cancer cases, but causes more than 80% of skin cancer deaths [1, 2]. In the USA, CMM ranks among the five most common malignancies [1, 3], and, over the past 50 years, its incidence and mortality rates have been increasing in all western countries, including Italy [4–8]. Data suggest that this increasing trend will continue in the coming decades [9–11].

People aged over 80 years are conventionally referred to as the “oldest-old” or “very old”. Between 2016 and 2050, the proportion of very old people will more than double worldwide, and this population will grow faster than the total population [12]. Optimizing clinical management of CMM in old patients today will mitigate problems anticipated to arise in the near future [13].

In CMM patients, age is an independent prognostic factor and previous studies have consistently associated CMM in old people with an unfavorable clinicopathological profile, including a higher prevalence of the nodular subtype, high Breslow thickness, high mitotic index, as well as a high prevalence of metastatic disease at clinical presentation [14–17].

Such a distinctive clinicopathological profile prompts dedicated primary and secondary prevention strategies, personalized diagnostic and therapeutic procedures, and age-tailored post-treatment follow-up schedules [18–20].

Fragmentary information is available on the epidemiological and clinical impact of CMM in old and very old patients [21–23]. This population-based study aims to provide a comparative analysis of the clinicopathological profile of CMM arising in adult, old, and very old patients.

## Methods

### Socio-epidemiological context

The Italian public healthcare system (PHS) is based on values of universality, free access, freedom of choice, pluralism in provision, and fairness. PHS is regionally managed and provides universal coverage supported by national taxation [24].

The Veneto is a north-eastern region of Italy with a resident population of 4,9 million (Females: 2,478,665; Males: 2,391,165; Mean age: 45.6 years).

In 2015, the Regional Oncology Network (Italian acronym: ROV) established clinical management procedures for oncology patients, including CMM. For each of the most incident malignancies, dedicated protocols recommend standardized clinical care pathways (Italian acronym: PDTA) covering prevention strategies, diagnostic-therapeutic procedures, and end-of-life care; specific indicators are also included to monitor consistency between the recommendations provided and real-world clinical practice [25–28].

### Regional cancer registry: high-resolution CMM recording

Since 2016, the Veneto cancer registry (Italian acronym; RTV) censors all malignancies occurring in the resident regional population. This population-based cohort study includes all incident cases of CMM recorded by RTV between January 1st and December 31st, 2017. Recording procedures rely on different information sources (e.g.: pathology reports, clinical records, death certificates, and health service administrative records) [29]. The CMM-related variables considered in this study include: sociodemographic data (sex and age categorized as < 65, 65–79, ≥ 80), primary CMM site (lower limbs, upper limbs, head, hands/feet, and trunk), CMM histotype (superficial spreading [SSM], nodular [N-CMM], lentigo maligna [LMM], acral-lentiginous, desmoplastic, Spitzoid melanoma, or not otherwise specified [NOS]), Breslow thickness (classified according to the AJCC 8th edition tumor categories [30] as ≤ 1, 1–2, 2–4, > 4 mm), Clark’s level of CMM spreading (I, II, III, IV, and V), CMM growth pattern (radial *versus* vertical), ulceration (absent *versus* present), mitotic count (number of mitoses per mm^2^), tumor-infiltrating lymphocytes ([TIL]; absent *versus* present), as well as T, N, and M AJCC stages at diagnosis (8th edition) [30].

### Indicators of clinical management

Based on the Manual of Melanoma Clinical Pathway Quality Indicators [27], and consistent with the recommendations of international scientific societies/institutions, the Veneto Regional Oncology Working Group (ROV) identified a set of clinicopathological indicators of consistency between recommended guidelines and regional oncology practice [31–38].

### Statistics

Categorical variables were described by their absolute frequency and percentage; the quantitative variable was described by median and interquartile range (Q1-Q3), since the Shapiro-Wilk normality test was rejected.

The association between age groups and categorical melanoma characteristics was investigated using a Chi-squared test or Fisher’s test. The latter was only used when there were less than five absolute frequencies in the contingency tables. When the null hypothesis (i.e., distribution is independent of age group) was rejected, a post-hoc analysis with Holm’s correction was performed for a pairwise comparison between age groups. The Kruskal-Wallis test was performed to test the independence between age groups and the quantitative variable, while the Mann-Whitney test was used for the following post-hoc analysis. Cramer’s V was also calculated to measure the strength of the association between each pair of variables. A diagram was produced in which the variables with a higher Cramer’s V value appeared closer together and were connected by darker, thicker lines. Variable pairs with a Cramer’s V value less than 0.1 were not connected. In this phase, the subjects with missing values in the variable considered to evaluate the association with age groups were excluded from the sample.

An ordered proportional odds logistic regression using age groups as the dependent variable was fitted using the anatomopathological characteristics of melanoma as covariates and correcting for sex, in order to test the association between age and the clinicopathological characteristics of melanoma in a multivariate setting. To avoid overadjustment, variables representing the presence of ulcerated lesions and melanoma thickness were not included in the explanatory variables, as they were already involved in the definition of melanoma stage. The cases with missing values were removed from the sample, reducing the sample size to 964.

Clinical performance indicators were computed (as percentages) for the three different age groups and their respective 95% confidence intervals (CI) were estimated using the Clopper-Pearson exact method. Independence tests (Chi-squared test or Fisher’s test) and post-hoc analysis (with Holm’s correction) were used to compare these values by age groups.

Results were deemed statistically significant at the *p* < 0.05 level. All statistical analyses were conducted using the computing software R 4.3.1.

### Ethics

This study project was formally approved by the Ethics Committee of the Veneto Oncological Institute (protocol number 52/2016). According to the study protocol, data analysis was conducted on anonymous aggregated data to minimize the chance of individuals being identified.

## Results

This study considered 1,368 incident CMM occurring throughout the regional population of Veneto between January 1st and December 31st, 2017. The “adult-group” included 779 (56.9%) patients, the “old-group” accounted for 408 (29.8%), and the remaining 181 (13.2%) were “very-old” (Table 1).

**Table 1:** Demographics and clinicopathological profile of the considered CMM patients

	Total		CMM Patients by age groups		*P* value ^i^			
	***N*** ** = 1,368**	< 65 *N* = 779 (56.9%)	65–79 *N* = 408 (29.8%)	≥ 80 *N* = 181 (13.2%)	All age groups	< 65 vs. 65–79	< 65 vs. ≥80	65–79 vs. ≥80
**Sex**								
Male	726 (53.1)	371 (47.6)	262 (64.2)	93 (51.4)	**< 0.001**	**< 0.001**	0.407	**0.009**
Female	642 (46.9)	408 (52.4)	146 (35.8)	88 (48.6)				
**Primary CMM site** ^**a**^								
Lower limbs	248 (18.9)	155 (20.4)	62 (16.2)	31 (18.1)	**< 0.001**	**< 0.001**	**< 0.001**	**< 0.001**
Upper limbs	172 (13.1)	95 (12.5)	46 (12.0)	31 (18.1)				
Head	151 (11.5)	47 (6.2)	68 (17.7)	36 (21.1)				
Hands/feet	63 (4.8)	25 (3.3)	19 (5.0)	19 (11.1)				
Trunk	680 (51.7)	438 (57.6)	188(49.1)	54 (31.6)				
**CMM histotype**								
Superficial spreading	948 (69.3)	591 (75.9)	269 (65.9)	88 (48.6)	**< 0.001**	**< 0.001**	**< 0.001**	**0.001**
Nodular	206 (15.1)	81 (10.4)	72 (17.6%)	53 (29.3)				
Lentigo maligna	32 (2.3)	8 (1.0)	14 (3.4%)	10 (5.5)				
Acral-lentiginous	23 (1.7)	11 (1.4)	5 (1.2%)	7 (3.9)				
Desmoplastic	7 (0.5)	1 (0.1)	3 (0.7)	3 (1.7)				
Spitzoid	30(2.2)	25 (3.2)	5 (1.2)	0				
CMM not otherwise specified	122 (8.9)	62 (8.0)	40 (9.8)	20 (11.0)				
**CMM thickness (Breslow)** ^**b**^								
≤ 1	787 (57.5)	513 (65.9)	210 (51.5)	64 (32.4)	**< 0.001**	**< 0.001**	**< 0.001**	**< 0.001**
1–2	204 (14.9)	131 (16.8)	53 (13.0)	20 (11.0)				
2–4	151 (11.0)	59 (7.6)	60 (14.7)	32 (17.7)				
> 4	139 (10.2)	39 (5.0)	49 (12.0)	51 (28.2)				
Median (Q1-Q3)	0.7 (0.4–1.7)	0.6 (0.4–1.2)	0.9 (0.4–2.3)	1.9 (0.6–4.9)	**< 0.001**	**< 0.001**	**< 0.001**	**< 0.001**
**Clark’s levels** ^**c**^								
I	3 (0.3)	2 (0.3)	1 (0.3)	0	**< 0.001**	**< 0.001**	**< 0.001**	0.096
II	328(28.6)	223 (33.1)	77 (23.7)	28 (19.2)				
III	427 (37.3)	283 (42.0)	105 (32.3)	39 (26.7)				
IV	338 (29.5)	155 (23.0)	122 (37.5)	61 (41.8)				
V	49 (4.3)	11 (1.6)	20 (6.2)	18 (12.3)				
**Growth pattern** ^**d**^								
Radial	270 (25.1)	183 (28.8)	69 (22.2)	18 (14.1)	**< 0.001**	0.073	**0.002**	0.073
Vertical	804 (74.9	452 (71.2)	242 (77.8)	110 (85.9)				
**CMM Ulceration** ^**e**^								
Present	251 (19.7%)	94 (12.7)	86 (23.1)	71 (43.3)	**< 0.001**	**< 0.001**	**< 0.001**	**< 0.001**
Absent	1023 (80.3)	644 (87.3)	286 (76.9)	93 (56.7)				
**Mitotic count per HPF** ^**f**^								
Median (Q1-Q3)	1 (0–3)	0 (0–2)	1 (0–4)	3 (0–8)	**< 0.001**	**0.001**	**< 0.001**	**< 0.001**
**TIL** ^**g**^								
Present	862 (73.2)	520 (76.5)	246 (70.7)	96 (64.4)	**0.005**	0.105	**0.01**	0.202
Absent	315 (26.8)	160 (23.5)	102 (29.3)	53 (35.6)				
**TNM Stage** ^**h**^								
I	905 (68.0)	588 (77.6)	239 (60.4)	78 (44.1)	**< 0.001**	**< 0.001**	**< 0.001**	**< 0.001**
II	218 (16.4)	72 (9.5)	80 (20.2)	66 (37.3)				
III	141 (10.6)	72 (9.4)	49 (12.4)	20 (11.3)				
IV	67 (5.0)	26 (3.4)	28 (7.1	13 (7.3)				

Data non-available in ^**a**^54 (3.9%), ^**b**^87 (6.4%), ^**c**^223 (16.3%), ^**d**^294 (21.5%), ^**e**^94 (6.9%), ^**f**^163 (11.9%), ^**g**^191 (14.0%), ^**h**^37 (2.7%). ^i^: in bold statistically significant values (*p* < 0.05). Acronyms: CMM: Cutaneous malignant melanoma; HPF: high power microscopic field; TIL: Tumor infiltrating lymphocytes; Q1: first quartile; Q3: third quartile

All the considered CMM clinicopathological variables differed significantly by patient age (Table 1). On comparing the three age-groups, significant differences emerged in CMM topography, prevalence of histotype and ulcer lesions, and CMM thickness. Moreover, the median mitotic count steadily increased by age (< 65 = 0; 65–79 = 1; ≥80 = 3) and older patients showed a significantly higher prevalence of vertical growth pattern and the lowest prevalence of tumor infiltrating lymphocytes (TIL). At initial diagnosis, the prevalence of TNM stage I was lowest among older patients and steadily increased by age group (44.1% *versus* 60.4% *versus* 77.6%).

**Fig. 1:**
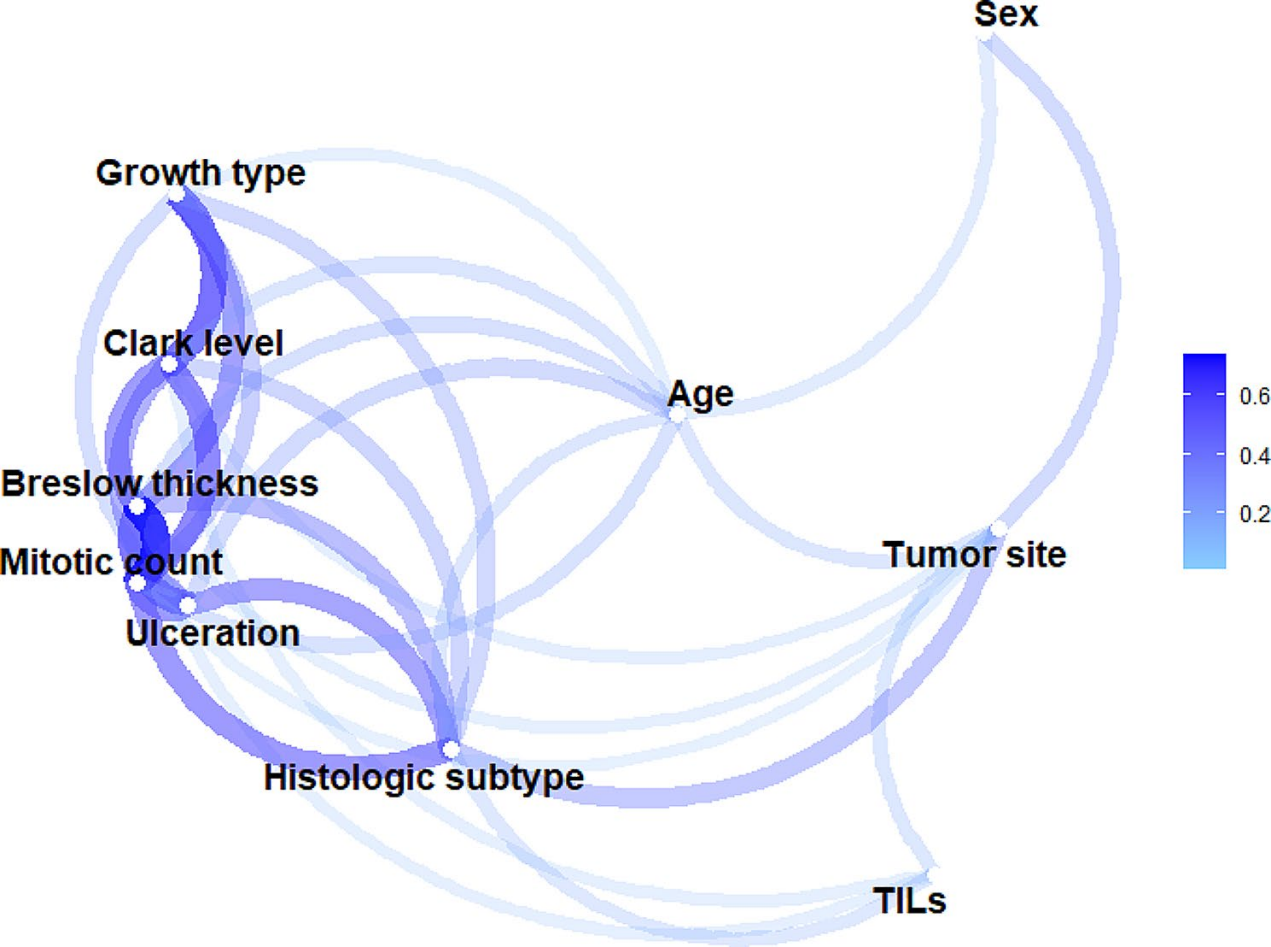
Pairwise association between anatomopathological and sociodemographic variables

Figure 1 shows the strength of pairwise associations between anatomopathological and sociodemographic variables of melanoma.

Multivariable ordered logistic regression (Table 2) confirmed the associations between age groups and tumor site and CMM stage at clinical presentation.

**Table 2:** Ordered proportional odds logistic regression model

	OR	95% CI	*p*-value ^a^
**Sex (reference: Female)**			
Male	1.61	1.22–2.11	**< 0.001**
**Tumor site (reference: Upper limbs)**			
Lower limbs	0.88	0.55–1.42	0.602
Head	1.98	1.19–3.30	**0.009**
Hands/feet	2.18	1.14–4.18	**0.019**
Trunk	0.68	0.45–1.01	0.058
**Histologic subtype (reference: Nodular melanoma)**			
Superficial spreading	0.85	0.54–1.33	0.470
Other	0.88	0.48–1.61	0.673
**Growth type (reference: Radial)**			
Vertical	1.11	0.78–1.57	0.555
**Mitotic count per high power microscopic field**			
	1.05	1.01–1.08	**0.011**
**Tumor infiltrating lymphocytes (reference: Present)**			
Absent	1.32	0.99–1.76	0.063
**TNM Stage (reference: stage I)**			
II	2.77	1.79–4.30	**< 0.001**
III	1.13	0.70–1.83	0.623
IV	1.40	0.55–3.58	0.482

^a^: in bold statistically significant values (*p* < 0.05). Acronyms: OR: odds ratio; CI: confidence interval

Table 3 focuses on the association between age groups and clinical performance indicators. The percentage of patients with 1–4 mm thick lesions admitted to sentinel lymph node biopsy (SLNB) decreased as age increased. Notably, fewer SLNB-positive patients underwent lymphadenectomy. The prevalence of TNM stage IB–III CMM patients treated with wide surgical excision who underwent nodal ultrasound within 12 months of CMM presentation was significantly lower in the ≥ 80s than in the other age groups.

**Table 3:** Clinical performance indicators by age groups

		Age < 64 years	Age 65–79 years	Age ≥ 80 years	*P*-value ^b^				N	%
INDICATOR	TH (%)^a^	% (95% C.I.)	% (95% C.I.)	% (95% C.I.)	All age groups	< 65 vs. 65–79	< 65 vs. ≥ 80	65–79 vs. ≥ 80		
Percentage of new cases of invasive CMM assessed for neoplastic ulcer	≥ 90	94.87 (93.07–96.31)	91.42 (88.27–93.95)	90.61 (85.39–94.43)	**0.024**	0.085	0.089	0.870	1,368	100.00
CMM-TNM stage I–IIA (%) undergoing head CT scans, chest CT/MRI scans, abdominal CT/MRI scans, or PET scans within 180 days after diagnosis	< 10	3.03 (1.81–4.75)	3.82 (1.85–6.91)	3.45 (0.72–9.75)	0.779	-	-	-	943	68.93
Percentage of patients with 1-4-mm thick lesions undergoing sentinel lymph node biopsy (SLNB)	≥ 90	94.16 (89.20-97.29)	81.63 (72.53–88.74)	60.00 (45.18–73.59)	**< 0.001**	**0.007**	**< 0.001**	**0.008**	1,066	77.92
Percentage of patients with lesions < 0.8 mm in thickness and no reported ulceration or mitoses undergoing SLNB	< 10	4.43 (2.44–7.32)	3.76 (1.23–8.56)	2.44 (0.06–12.86)	1.000	-	-	-	490	35.82
Percentage of patients with time elapsing between biopsy and complete excision < 60 days	≥ 90	62.27 (58.62–65.83)	59.77 (54.45–64.93)	58.47 (49.04–67.47)	0.600	-	-	-	1,192	87.13
Percentage of cases with pT1-T2 disease ≤ 2.0 mm in thickness and surgical margins < 0.8 cm	< 10	31.74 (27.99–35.68)	26.07 (20.57–32.19)	32.84 (21.85–45.40)	0.251	-	-	-	887	66.59
Percentage of cases with pT1, pT2 disease ≤ 2.0 mm in thickness and surgical margins > 1.2 cm	No-TH	24.23 (20.81–27.91)	30.34 (24.52–36.67)	38.81 (27.13–51.50)	**0.015**	0.173	**0.044**	0.247	887	66.59
Percentage of cases with pT3, pT4 disease 2.0 mm in thickness and surgical margins < 1.6 cm	< 10	57.50 (45.94–69.78)	59.30 (48.17–69.78)	69.57 (54.25–82.26)	0.381	-	-	-	212	15.69
Percentage of cases with pT3, pT4 disease > 2.0 mm in thickness and surgical margins > 2.4 cm	No-TH	3.75 (0.78–10.57)	6.98 (2.60-14.57)	4.35 (0.53–14.84)	0.727	-	-	-	212	15.69
Percentage of SLNB-positive patients	≥ 15	18.01 (14.18–22.37)	18.85 (13.56–25.13)	16.36 (7.77–28.80)	0.911	-	-	-	607	44.37
Percentage of SLNB-positive patients undergoing lymphadenectomy	No-TH	85.45 (73.34–93.50)	84.38 (67.21–94.72)	44.44 (13.70–78.80)	**0.024**	1.000	0.039	0.051	96	7.02
Percentage of patients undergoing SLNB in a regional reference center	≥ 90	62.88 (57.67–67.88)	59.38 (52.07–66.39)	50.00 (35.81–64.19)	0.187	-	-	-	605	44.23
Percentage of TNM stage IB–III patients undergoing nodal US within 12 months of wide excision	≥ 95	61.69 (56.41–66.77)	62.38 (55.31–69.08)	38.64 (28.44–49.62)	**< 0.001**	0.945	**< 0.001**	**< 0.001**	645	48.24

^a^ No-TH were established in the absence of supporting scientific evidence. ^b^: in bold statistically significant values (*p* < 0.05)

Acronyms: TH = thresholds; CMM: Cutaneous malignant melanoma; SLNB: sentinel lymph node biopsy

## Discussion

This population-based cohort study compared the clinicopathological features of CMMs at clinical presentation in a large cohort of northern-Italian CMM patients stratified by age (i.e., adult, old, and very old). Compared to adult and old patients, the older subjects displayed a distinct disease profile in terms of gender balance, tumor topography, higher prevalence of neoplastic ulcer, a more aggressive pattern of local spreading, decreased TIL, and advanced TNM stages.

### CMM histotype and local spreading

Among older patients, the prevalence of nodular-CMM was significantly higher than recorded in patients of adult age. This histotype-dependent aggressiveness is consistent with the high prevalence of epidermal invasion (resulting in neoplastic ulcer) and deep cutaneous spreading (resulting in high Breslow thickness and Clark’s levels). Conversely, superficial spreading melanoma significantly prevailed in adult and old patients, providing the biological rationale for the less aggressive CMM behavior associated with the younger study population.

Age-related prevalence of histological subtypes was a key determinant of the different CMM stages at clinical presentation. Compared to adults, old and very old patients showed a significantly higher prevalence of advanced stages [21, 22], representing the most well-established adverse prognostic variable.

Moreover, in older patients, the invasive behavior of the nodular histotype may be exacerbated by declining age-related immunocompetence [39]. Indeed, the study findings suggested an association between older CMM patients and a lower prevalence of tumor infiltrating lymphocytes (TIL) [40], which is a reliable indicator of the host’s immunoreaction against melanomatous cells [40–42].

### Timely diagnosis in older patients

In old and (more so) in very old patients, the advanced CMM stage at presentation plausibly resulted from a combination of greater CMM aggressiveness and diagnostic delay [43]. A declining interest in personal care (particularly skin self-examination), susceptibility to depression and mood disorders, decreased family/social support and, more in general, age-related frailty, may explain the diagnostic delay [44, 45].

In this peculiar setting, the involvement of general practitioners in the diagnosis, hopefully supported by “virtual” tele-dermatology, may provide “at-home” monitoring of at-risk lesions, ultimately promoting secondary prevention strategies [46]. A Cochrane systematic review found that more than 93% of malignant skin lesions may be confidently assessed by tele-dermatology [47], and this digital opportunity may play a crucial role in diagnostic anticipation.

### Diagnostic-therapeutic workup in old CMM patients

Sentinel nodal biopsy (SLNB), lymphadenectomy (in SLNB-positive cases), and ultrasound investigation of nodal status were applied less in older than in younger patients. Similar results have been reported elsewhere and were interpreted as resulting from patient comorbidities or older patients’ poor compliance with aggressive treatments [18–21]. Moreover, the impact of SLNB on old patients’ survival was not documented, thus lowering the clinical priority of the “sentinel” procedure [18] and prompting the need for personalized diagnostic/therapeutic readjustments to balance effective cancer therapy with appreciable quality of life.

The present results also show that old patients often underwent “extended” CMM surgical excision, theoretically prioritizing patient safety over esthetic expectancies. The ethical and clinical implications of these therapeutic choices warrant more extensive investigation [17, 48–50].

The main strength of this study is its population-based (rather than center-specific) design, thus minimizing the risk of selection bias. Moreover, the use of standardized algorithms reduced measurement variability, thereby increasing the reliability of the values.

In terms of limitations, first, the lack of some variables (e.g., CMM molecular profiling) could have led to important differences being missed in each of the CMM age-groups. Second, the study is limited to the 2017 data, since more recent and complete data were not available for the analysis.

## Conclusion

In older patients, the clinicopathological presentation of CMM differs from that of general population. Compared to malignancies at a younger age, older patients showed a higher prevalence of the head, hands, or feet as the primary site, and a higher TNM stage at presentation.

Clinical management also differs, with less frequent SLNB biopsies and lymphadenectomy (in SLN-positive cases). In all cases, but particularly in older frail patients, tele-dermatology could efficiently activate secondary prevention strategies [51].

## Data Availability

The data supporting this study’s findings are held by the Veneto Epidemiological Registry and were used under license for this work. The anonymized minimal data set necessary to replicate our findings have been made publicly available at the following link: 10.6084/m9.figshare.24961311.

## Abbreviations

CMM: Cutaneous malignant melanoma
PHS: Italian public healthcare system
RTV: Veneto cancer registry
SLNB: Sentinel lymph node biopsy
TIL: Tumor-infiltrating lymphocytes
